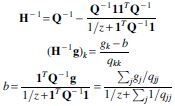# Modeling Multiplexed Images with *Spatial-LDA* Reveals Novel Tissue Microenvironments

**DOI:** 10.1089/cmb.2019.0340

**Published:** 2020-08-04

**Authors:** Zhenghao Chen, Ilya Soifer, Hugo Hilton, Leeat Keren, Vladimir Jojic

**Affiliations:** ^1^ Calico Life Sciences LLC, South San Francisco, California, USA.; ^2^ Department of Pathology, Stanford University, Stanford, California, USA.

**Keywords:** cellular microenvironment, in situ multiplexed imaging, LDA, spatial profiling, topic models

## Abstract

**Recent in situ multiplexed profiling techniques provide insight into microenvironment formation, maintenance, and transformation through a lens of localized cellular phenotype distribution. In this article, we introduce a method for recovering signatures of microenvironments from such data. We use topic models to identify characteristic cell types overrepresented in neighborhoods that serve as proxies for microenvironment. Furthermore, by assuming spatial coherence among neighboring microenvironments our model limits the number of parameters that need to be learned and permits data-driven decisions about the size of cellular neighborhoods. We apply this method to uncover anatomically known structures in mouse spleen—identifying distinct population of spleen B cells that are defined by their characteristic neighborhoods. Next, we apply the method to a dataset of triple-negative breast cancer tumors from 41 patients to study the structure of tumor-immune boundary. We uncover previously reported tumor-immune microenvironment near the tumor-immune boundary enriched for immune cells with high Indoleamine-pyrrole 2,3-dioxygenase (IDO) and Programmed death-ligand 1 (PD-L1) and a novel, immunosuppressed, microenvironment-enriched for cells expressing CD45 and FoxP3.**

## 1. Introduction

Human tissues need multiple cell types and complex organization to function. Single-cell transcriptome and chromatin profiling provide an unprecedented resolution of the complexity of tissue compositions and how it changes as a result of various genetic or environmental perturbations. However, because these methods require dissociation of the tissue into single cells, they lack the ability to resolve the structure of the tissues. Moreover, single-cell profiling reveals significant heterogeneity in transcriptional state even within a single-cell type. How this heterogeneity is affected by or affects the interactions that the cell undergoes with other cells is largely unknown, but numerous examples of the effect of cellular environment on cellular function exist. In this study, we focus on identifying microenvironments in the context of interactions between elements of the immune system.

The ability of the immune system to mount an effective response is increasingly thought to be dependent on composition of the immune environment within a tissue or tumor. These immune microenvironments are defined by their cell types, spatial organization, biochemical signals, cell–cell and receptor–ligand interactions, whose coordination regulates the migration, differentiation, and response of immune cell subsets, and, ultimately, the success or failure of an organism to recognize and remove malignant cells or an invading pathogen.

The tumor immune microenvironment (TME) is now recognized as a critical determinant of patient outcome (Galon et al., 2006; Bindea et al., 2013). The exclusion of tumor-infiltrating lymphocytes (TILs) from the vicinity of cancer cells is negatively correlated with survival (Galon et al., 2013) and an understanding of the immunosuppressive factors that drive this exclusion is an area of intense focus, particularly in the context of understanding the high rate of failure of immune checkpoint blockade therapy (Pitt et al., 2016).

Specialized immune microenvironments also play a critical role in the normal functioning of lymphoid organs such as the thymus (Ritter and Palmer, 1999). Here, interactions between various cell types govern the development of functionally mature naive T cells. Although the underlying mechanisms remain unclear, with increasing age, these thymic microenvironments become disrupted, their resultant disorder contributing to thymic atrophy and decline in naive T cell production (Aw et al., 2008). Similar disruption to the local microenvironment are observed in other aging immune organs such as the lymph nodes and are thought to contribute to immune deficiencies that accompany aging (Thompson et al., 2017).

Taken together, these studies highlight that effective immune responses are not simply dependent on the number or type of cells resident in a given tissue, but also their spatial organization, which show evidence of being disrupted with immune-mediated disease, increasing age, or in cancer.

## 2. Methods

### 2.1. In situ profiling of tissues and microenvironments

**FIG. 1.**
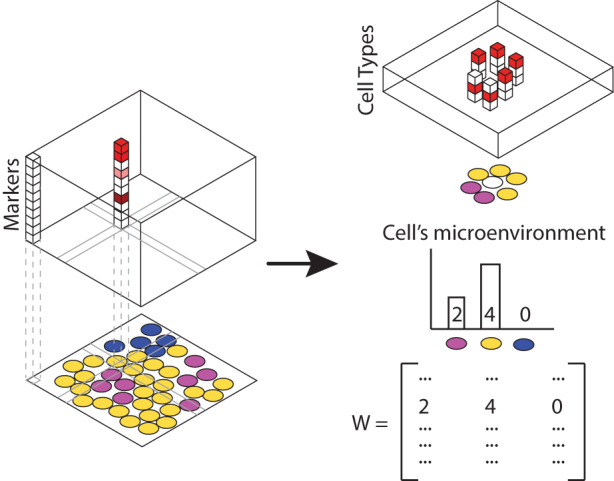
In situ profiling of tissue slices using technologies such as CODEX and MIBI-TOF enable simultaneous spatial measurement of a panel of markers. These markers can be aggregated into cellular phenotype. Counts of cell neighbor's phenotypes are proxies for cell microenvironment.

Novel in situ profiling technologies, such as “co-detection by indexing” (CODEX) (Goltsev et al., 2018) and “multiplexed ion beam imaging by time-of-flight” (MIBI-TOF) (Keren et al., 2018), enable detailed characterization of cellular organization in tissues—how various cell types are situated relative to each other. In both methods, a tissue section is imaged and at each location, the abundance of 30–40 markers of interest is measured. Cells can then be classified into various canonical cell types or characterized by the presence or absence of markers. This distribution of cellular phenotypes within a neighborhood carries information about the local microenvironment. In our approach we will use the local distributions of cell types or marker expression as a proxy for the microenvironment (Fig. 1).

### 2.2. Modeling of cellular distributions: Bag-of-cells

The key modeling assumption we make is that the organization of a local cellular neighborhood is invariant to reordering of cells. The rationale for this is twofold. First, a pair of cells in a sufficiently small neighborhood can easily signal to each other either through receptor binding or by signaling molecule secretion. Consequently, specific layout of cells is not critical to distinguishing microenvironments. Second, the number of equivalent multicellular patterns under rotations, mirroring, and translation is vast. Trying to learn all those patterns would necessitate models with large number of parameters, with all the associated disadvantages (e.g., computation time and overfitting). Consequently, we take a “bag-of-cells” approach similar to the “bag-of-words” idea in natural language processing and computer vision, where cell-type counts are used to represent a specific microenvironment.

### 2.3. Modeling a bag-of-cells

We built our model based on latent Dirichlet allocation (LDA) (Blei et al., 2003). This model is typically introduced using the documents-words-topics paradigm. We state the model in this paradigm, before mapping it to our domain. A text document, viewed as an unordered bag of words, is represented by word counts. Variable *w_ij_* is identity of word *j*th word in document *i*. Each word is latently associated with a topic, indicated by variable *z_ij_*. Topics are defined by their preference for specific words, parameter *β*. Each document has a topic preference *θ_i_* that is distributed with a Dirichlet prior parameterized by α. Compactly, LDA can be stated as

(1) $$\theta_{i}∼Dirichlet\left(\alpha \right).$$

(2) $$\beta_{k}∼Dirichlet\left(\eta \right).$$

(3) $$z_{ij}∼Multinomial\left(\theta_{i}\right).$$

(4) $$w_{ij}∼Multinomial\left(\beta_{z_{ij}}\right).$$

In our application, a “document” is composed of all cells in a small neighborhood. Words correspond to the phenotype of a cell. A topic is a cellular phenotype distribution associated with typical microenvironments.

The key tasks in this model are as follows:

- Learning of typical microenvironments.
- Inferring local microenvironment loadings.

Both these tasks can be seen as inference in a Bayesian model. For completeness, we describe a variational inference-based approach to solving these two tasks (Hoffman et al., 2010). This approach starts by forming an Evidence Lower BOund (ELBO):

$$\begin{array}{cc} & \log p\left(w|\alpha ,\eta \right)\geq E_{q}\left[\log p\left(w,z,\theta ,\beta |\alpha ,\eta \right)\right]-\\ & E_{q}\left[\log q\left(\theta ,z,\beta \right)\right] \end{array}.$$

With a factorization assumption, referred to as mean field,

$$q\left(\theta ,z,\beta \right)=\prod_{i}q\left(\theta_{i}\right)q\left(z_{i}\right)\prod_{k}q\left(\beta_{k}\right).$$

Factors of the posterior are given as

$$\begin{array}{cc} & q\left(z_{ij}=k\right)=\phi_{iw_{ij}k}\\ & q\left(\theta_{i}\right)=Dirichlet\left(\theta_{i};\gamma_{i}\right)\\ & q\left(\beta_{k}\right)=Dirichlet\left(\beta_{k};\lambda_{k}\right) \end{array}$$

ELBO optimization procedure iterates updates:

$$\begin{array}{cc} & \phi ={\mathrm{argmax}}_{\phi }E_{q}\left[\log p\left(w,z,\theta ,\beta |\alpha ,\eta \right)\right]\\ & -E_{q}\left[\log q\left(\theta ,z,\beta \right)\right] \end{array}.$$

$$\begin{array}{cc} & \gamma ={\mathrm{argmax}}_{\gamma }E_{q}\left[\log p\left(w,z,\theta ,\beta |\alpha ,\eta \right)\right]\\ & -E_{q}\left[\log q\left(\theta ,z,\beta \right)\right] \end{array}.$$

$$\begin{array}{cc} & \lambda ={\mathrm{argmax}}_{\lambda }E_{q}\left[\log p\left(w,z,\theta ,\beta |\alpha ,\eta \right)\right]\\ & -E_{q}\left[\log q\left(\theta ,z,\beta \right)\right]. \end{array}.$$

Upon convergence approximate posterior on *β*, *θ*, and *z* can be interrogated to provide topic definition, document topic preferences, and word-level topic assignments.

The LDA model assumes that documents are both reasonably long, and also independent of each other given the topic model's parameters.

In our context, the microenvironments are potentially occupied by a small number of cells—short documents. Furthermore, locally proximal cells are likely, although not guaranteed, to have similar microenvironments.

### 2.4. Spatially coherent bags-of-cells

This motivates an extension to the LDA model—to promote coherence of microenvironments between nearby cells, we introduce a prior on α, microenvironment preferences:

$$p\left(\alpha \right)\propto \prod_{\left(i,j\right)\in Edges}Laplace\left(\alpha_{i}-\alpha_{j};d_{ij}\right).$$

Here, edges denotes a set of tuples (*i*, *j*) denoting “adjacent” cells that are likely to share similarity in their microenvironment. In practice, there are several ways to induce an edge set based on the positions of a set of cells, such as using the *K*-nearest neighbor graph or connecting cells within a certain radius. In our following experiments, we induce an edge set by computing the Voronoi partitioning of cell positions and connect cells that share a facet in the Voronoi partitioning.

**FIG. 2.**
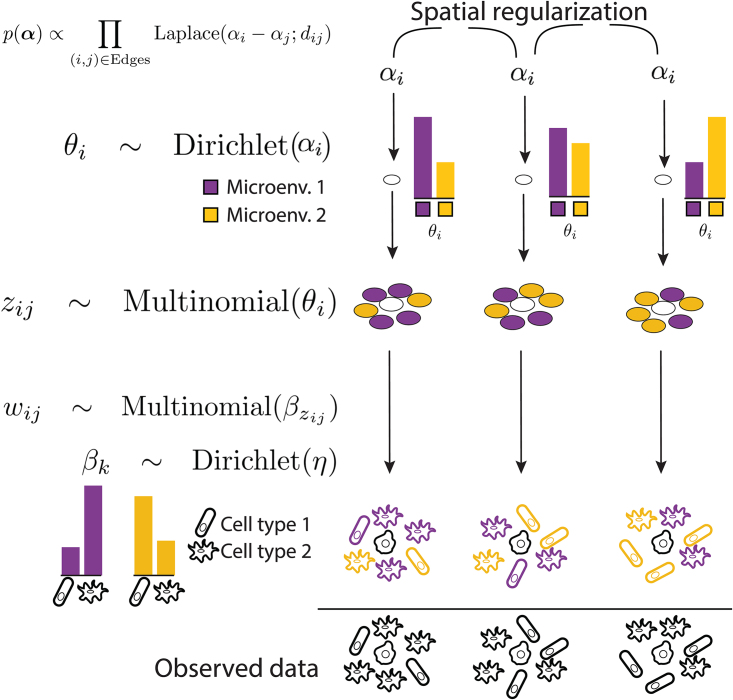
We introduce a model that ties together inferred microenvironments of nearby cells, thereby boosting the power to detect subtle microenvironmental changes. This assumption is encoded in similarity of as—previous preference for microenvironment. We anchor microenvironment to a cell shown in white. We consider two topics, purple and yellow. A particular neighborhood is a mixture of cells drawn from the two microenvironments. Variable *z* indicates whether a particular cell in the neighborhood was drawn from purple or yellow topic. *w*, a cell's phenotype (rod or flagellate), is drawn according to microenvironment's preference (e.g., purple microenvironment prefers flagellate). The observed information is only the shape (rod or flagellate), a cellular phenotype readout available from markers.

A schematic of the complete model is given in Figure 2. Henceforth, we will refer to this model as the spatial LDA model to distinguish it from the usual LDA topic model.

To incorporate this prior into model and training procedure, we rewrite the ELBO,

$$\begin{array}{cc} & \log p\left(w|\alpha ,\eta \right)\geq E_{q}\left[\log p\left(w,z,\theta ,\beta ,\alpha |\eta \right)\right]-\\ & E_{q}\left[\log q\left(\theta ,z,\beta ,\alpha \right)\right] \end{array},$$

where we assume

$$q\left(\theta ,z,\beta ,\alpha \right)=\prod_{i}q\left(\theta_{i}\right)q\left(z_{i}\right)\prod_{k}q\left(\beta_{k}\right)q\left(\alpha \right)$$

and

q(α)=δ(α−ξ).

Simplification of the above bound to terms that involve only *ξ* leads to

$$\begin{array}{cc} & B\left(\xi \right)=\sum_{i}\left[\log\frac{\Gamma \left(\sum_{k}\xi_{ik}\right)}{\prod_{k}\Gamma \left(\xi_{ik}\right)}+\sum_{k}\xi_{ik}c_{ik}\right]\\ & -\sum_{\left(i,j\right)\in Edges}\frac{1}{d_{ij}}\left|\xi_{i}-\xi_{j}\right|, \end{array}$$

where

$$c_{ik}=\Psi \left(\gamma_{ik}\right)-\Psi \left(\sum_{k}\gamma_{ik}\right),$$

and we obtain updates

$$\begin{array}{cc} & \phi ,\gamma ,\lambda ={\mathrm{argmax}}_{\phi ,\gamma ,\lambda }E_{q}\left[\log p\left(w,z,\theta ,\beta |\alpha =\xi ,\eta \right)\right]\\ & -E_{q}\left[\log q\left(\theta ,z,\beta \right)\right] \end{array}$$

$$\xi ={\mathrm{argmax}}_{\xi }B\left(\xi \right).$$

We optimize the ELBO by alternating between the first update, which only requires a slight modification to any existing LDA library and the second update of *ξ*. However, the update of *ξ* involves optimizing a nonsmooth function [Equation (5)] across thousands of cells per sample. To do this efficiently, we use an alternating direction method of multipliers (ADMM) (Boyd et al., 2011) + primal-dual interior point optimization approach (Boyd and Vandenberghe, 2004) we refer the reader to the Appendix for details and the full derivation of our method.

The spatial LDA model introduces a new free parameter *d_ij_*, which inversely correlates with how strongly we believe cells *i* and *j* are similar in their topic preferences. In other words, the smaller *d_ij_* is, the more strongly we constrain adjacent cells *i* and *j* to have equal topic preferences.

## 3. Results and Discussion

### 3.1. Topic modeling identifies fine grained structures in mouse spleens

We first applied our framework to identify cellular microenvironments of B cells in mouse spleen. The spleen is a heterogeneous but highly structured organ that contains multiple resident cell types that makes it a good validation model. A previous study had acquired images of z-sections of mouse spleens from normal and diseased mice, each stained with a panel of 30 different antibodies using CODEX that we use in our experiments hereunder (Goltsev et al., 2018).

**FIG. 3.**
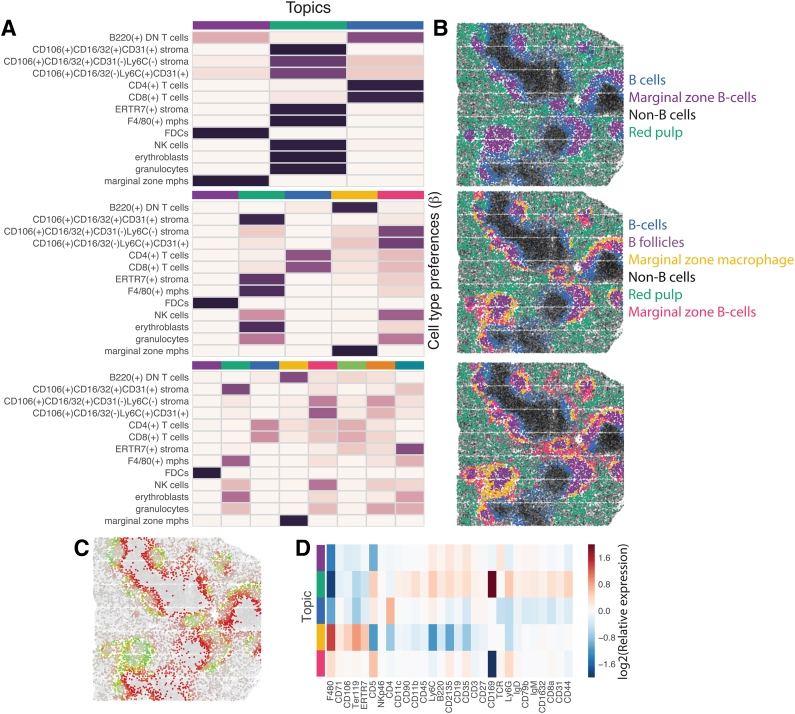
Spatial LDA reveals characteristic neighborhoods of B cells in mouse spleen. **(A)** Row-normalized cell type preferences of the topics fitted to the data by spatial LDA assuming 3, 5, and 8 topics. **(B)** Wild-type sample 1 from Goltsev et al. (2018) where each B cell is colored according to its main topic assuming 3, 5, and 8 topics. Note increasing resolution of the structures with increasing number of topics. Black denotes non-B cells. **(C)** Smooth transition between topic weights in spleen. Shown are the weights of topics 3 and 4 in five-topic model in the same sample as in (B). **(D)** Distinct gene expression profiles of B cells in different neighborhoods. Normalized (Log2) average expression of each marker in each topic for spatial LDA model with five topics. LDA, latent Dirichlet allocation.

We first asked if our technique identified distinct microenvironments that affect the state of B cells. We chose B cells as they are very abundant within the spleen and extensive literature exists regarding their distinct subpopulations in different locations of the spleen. The CODEX dataset contains images of three wild-type spleens with cell-type annotations. To generate input for the spatial LDA model, for every B cell in the dataset, we generated a vector of cell type counts of its non-B cell neighbors within a 3D ball of radius 100 pixels. We then applied spatial LDA on this vectors to generate an increasing number of topics (Fig. 3A).

#### 3.1.1. Spatial LDA enables the characterization of microenvironment at different scales

Increasing the number of fitted topics allowed us to probe the spatial organization of the cellular microenvironment with increasing resolution. Fitting the spatial LDA model with three topics resolved only differences between the largest regions of the spleen, namely white pulp B cells and the marginal zone B cells. Fitting five topics revealed a finer structure of follicular B cells, and two types of marginal zone B cells (macrophage associated and a stromal subset associated with natural killer (NK) cells and granulocytes) (Fig. 3B).

This also suggests an intuitive strategy for deciding the number of topics to fit—one can vary the number of topics depending on the level of granularity that is required for analyzing a dataset of choice, potentially increasing the number of topics until topics are no longer consistently reproduced run-to-run.

#### 3.1.2. Spatial LDA captures smoothly transitioning microenvironments

Another natural approach to identifying characteristic neighborhoods is by clustering cell-type counts, an approach taken in Goltsev et al. (2018). However, a clustering model is a bad choice at capturing boundary transitions between two microenvironments. Our approach, on the contrary, allows for gradual transition between different microenvironments, as each neighborhood is modeled as a combination of topics. For example, transition between white pulp and marginal zone B cells or between marginal zone and the red pulp B cells is gradual, as reflected by the continuous transition in the topic weights in Figure 3C.

#### 3.1.3. Topics learned by spatial LDA are biologically consistent

Although spatial LDA identified multiple topics with distinct localization patterns, it was not clear if these are indeed biologically distinct subpopulations of B cells. To answer this question, we had a trained immunologist label each topic with a label based only on the spatial distribution of that topic within the spleen. We then looked at the average expression of all measured markers as grouped by microenvironment topic (Fig. 3D) and found that each microenvironment had a characteristic expression pattern consistent with known biology. For example, identification of the follicular B cell topic was made on the basis of its characteristic outline and concentration at the periphery of an area identified as white pulp. Follicular B cells are surrounded by a complex network of mesenchymal follicular dendritic cells. This expected association was seen in high expression of the follicular dendritic cell marker CD21/35 in this microenvironment.

Similarly, the splenic red pulp typically contains F8/80 expressing macrophages that play an important role in red blood cell homeostasis, which we also observe in the topic weights of our subset identified as red pulp.

### 3.2. Topic modeling identifies clinically relevant tumor-immune microenvironment topics

Characterizing the spatial organization of the TME is of interest in cancer biology because of the complex interactions between tumor cells and immune cells that are known to influence response to treatment and survival (Galon et al., 2006; Bindea et al., 2013; Pitt et al., 2016). In previous study, Keren et al. (2018) collected and analyzed a dataset consisting of 41 triple-negative breast cancer tumors using MIBI-TOF and classified tumors into cold, mixed, and compartmentalized subsets corresponding to increasing degrees of intermixing between tumor and immune cells. In particular, they found that compartmentalized tumors were characterized by a clear tumor-immune boundary and was associated with better survival.

As further validation of our framework, we applied the spatial LDA model to this dataset of triple negative breast cancer tumors from Keren et al. (2018). We defined the immune neighborhood of a tumor cell as the count of all immune cells within a 39 μm (100 pixels) radius of the cell center. We then generated a histogram of 36 counts—each count representing the number of immune cells in a neighborhood expressing a given cell marker—and applied the spatial LDA model to learn five TME topics. To summarize the topic distribution for a tumor, we compute the fraction of tumor cells that have topic weight >1/number of topics for a given topic across all topics.

#### 3.2.1. Spatial LDA identifies two tumor-immune microenvironments near the tumor-immune boundary

Previous work Keren et al. (2018) proposed a method for identifying the tumor-immune boundary by smoothing the density of immune and tumor cells and aggregating them into connected components. In our study, we replicate their findings, demonstrating that the tumor-immune boundary is characterized by a distinct TME that can be inferred directly from the local composition of immune cells.

**FIG. 4.**
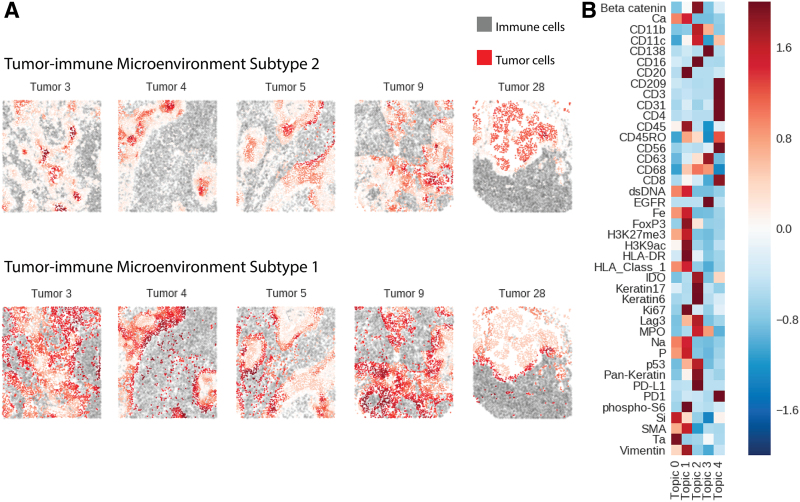
**(A)** Two TME topics found near the tumor-immune boundary. Topic 2 corresponds to the TME cluster reported in Keren et al. (2018), whereas topic 1 is a new, immunosuppressive topic. Red points denote tumor cells where intensity denotes the degree to which a tumor cell's microenvironment resembles a given topic. Gray points denote immune cells. **(B)** Topics discovered by spatial LDA and their preferences for cells expressing different markers. Red entries denote a strong preference for cells expressing that marker and blue relatively low preference. TME, tumor immune microenvironment.

We identified two distinct TME topics near the tumor-immune boundary (Fig. 4a). Our first TME topic (topic 2) corresponded to the tumor-immune boundary TME reported by Keren et al. (2018); immune cells in this region coexpressed high levels of Indoleamine-pyrrole 2,3-dioxygenase (IDO), Programmed death-ligand 1 (PD-L1), Integrin alpha M (CD11b), and Integrin alpha X (CD11c) (Fig. 4b). This TME topic generally lies close to but not directly on the tumor-immune boundary [Fig. 4a or Fig. 6 in Keren et al. (2018)]. However, we also identify a second TME topic (topic 1), which typically lies much closer to or on the tumor-immune boundary itself. In this second TME topic, immune cells express high levels of CD45, and FoxP3—possibly indicating the presence of immunosuppressive regulatory T cells.

In our survival analysis, the presence of TME topic 2 was associated with better survival even after stratifying on compartmentalized versus mixed tumors (Fig. 6). In contrast, TME topic 1 was not significantly associated with overall survival.

#### 3.2.2. Spatial LDA identifies substructure within tumor interior and mixed tumors

**FIG. 5.**
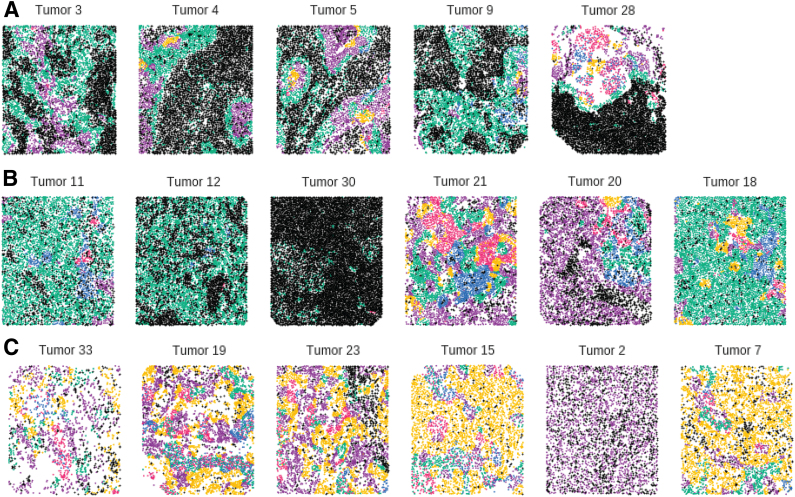
Tumor sections colored by the “dominant” TME topic (TME topic with the highest weight) for each cell. Note the heterogeneity within the tumor interior and across the mixed tumor samples. Purple corresponds to topic 1, green to topic 2, blue to topic 3, yellow to topic 4, and pink to topic 5. Note the overrepresentation of TME topic 4 in mixed tumors with poor prognosis. **(A)** Five compartmentalized tumors. **(B)** Bottom quartile of mixed tumors by predicted hazard. **(C)** Top quartile of mixed tumors by predicted hazard.

In addition, we identify two TME topics found in the interior of tumors (Fig. 5A).

**FIG. 6.**
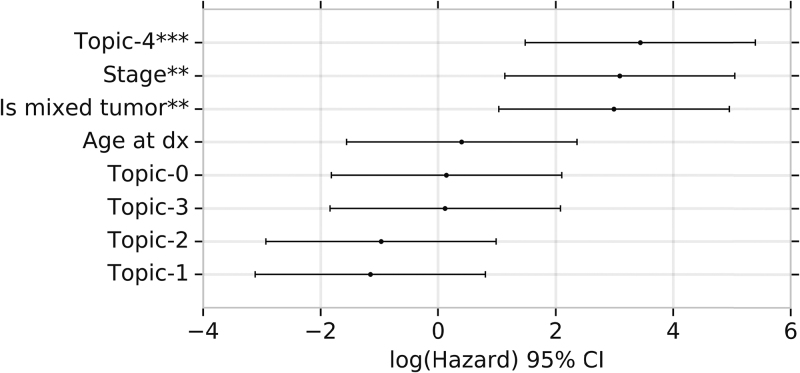
Cox regression coefficients [expressed as log(hazard ratio) with 95% confidence interval] of regressing overall survival, across all tumors (both mixed and compartmentalized), on proportion of tumor cells in a particular TME topic (controlling for mixed vs. compartmentalized tumors, stage, and age at diagnosis). **Significant at <0.01 level. ***Significant at <0.001 level.

We identify a TME topic (topic 3) that is typically located deep within the interior of compartmentalized tumors (colored yellow in Fig. 5A–C) characterized by the lack of immune cells expressing CD8, CD45 consistent with a dearth of infiltrating cytotoxic T lymphocytes (TILs). Confirming this finding, the average proportion of TME topic 3 within a tumor is also strongly negatively correlated with TIL score (*p* < 0.005, Spearman rank test). TME topic 3 is also strongly overrepresented in mixed tumors with poor predictive survival (Fig. 5B, C), but the proportion of tumor cells in a TME topic 3-like microenvironment is not significantly associated with poor survival after controlling for mixed status (Fig. 6).

We further identified a distinct TME topic (topic 4) also typically found in the interior of compartmentalized tumors (colored pink in Fig. 5A–C) characterized by high proportion of immune cells expressing CD3, CD4, CD8, CD45RO, and PD1. The proportion of tumor cells with a TME topic 4-like microenvironment is also strongly associated with poor overall survival and we hypothesize that this TME topic represents an immunosuppressed TME because of the high expression of PD1 and CD45RO. TME topic 4 is the most negatively associated with survival out of all the TMEs identified by spatial LDA (Fig. 5).

## 4. Conclusion

The advent of in situ multiplexed imaging techniques such as CODEX (Goltsev et al., 2018) and MIBI-TOF (Keren et al., 2018) enable the quantification of dozens of molecular markers at subcellular resolution. This motivates the development of analytical tools that model such data.

In this article, we present a model of cellular microenvironment called spatial LDA. We extend the well-known LDA model by introducing a regularization term that encourages agreement about microenvironments between nearby cells. We also derive an efficient variational Bayes update procedure to fit such models, alternating between fitting an almost standard LDA model and an ADMM + primal-dual interior point optimization to update the topic prior. Spatial LDA is able to model smooth transitions between microenvironments, captures organization at multiple scales, and increases power to infer microenvironment types using positional information.

To validate the effectiveness of spatial LDA, we apply spatial LDA to two existing datasets, one of mouse spleens (Goltsev et al., 2018) and one of Triple-negative breast cancer (TNBC) tumors (Keren et al., 2018).

We validate our model by recovering known immunological compartments in mouse spleen (Goltsev et al., 2018) and identifying clinically relevant microenvironments in TNBC (Keren et al., 2018).

We find that spatial LDA is able to identify distinct subpopulations of B cells in the mouse spleen at multiple scales and capture gradual transitions in the microenvironment. These subdivisions of B cells identified also reflect known biology of B cell compartments in the spleen.

When applied to a dataset of TNBC tumors, spatial LDA is able to recover previously reported features of the TME near the tumor-immune boundary. In addition, it also identified several novel TME types both within the tumor interior and along the tumor-immune boundary.

We hope that spatial LDA provides both a tool for analyzing tissue microenvironments and a foundation on which more complex topic models can be developed.

## 5. Appendix—Deriving Updates for Topic Prior

### 5.1. Spatially regularized LDA

We extend the usual LDA model such that each document has a topic prior *α_i_* and introduce a prior on *α* = (*α_1_*_,_…, *α_n_*) and an edge set (Edges) connecting “neighboring” cells.

$$p\left(\alpha \right)\propto \prod_{\left(i,j\right)\in Edges}Laplace\left(\alpha_{i}-\alpha_{j};d_{ij}\right),$$

where *d_ij_* is a constant or a deterministic function of a spatial distance between *i* and *j*. The variational lower bound (ELBO) then becomes

$$L\left(\phi ,\gamma ,\lambda ,\xi \right)=E_{q}\left[\log p\left(w,z,\theta ,\beta |a,\eta \right)p\left(\alpha \right)\right]-E_{q}\left[\log q\left(\theta ,z,\beta ,\alpha \right)\right].$$

We will assume

$$q_{\xi }\left(\alpha \right)=\delta \left(a-\xi \right).$$

Considering only terms involving ***α*** and noting that entropy is 0 for delta function:

$$E_{q}\left[\log p\left(\theta |\alpha =\xi \right)\right]+\log p\left(\alpha =\xi \right)=E_{q}\left[\log p\left(\theta |\alpha =\xi \right)\right]-\sum_{\left(i,j\right)\in Edges}\frac{1}{d_{ij}}\left|\xi_{i}-\xi_{j}\right|.$$

We plug in distributions

$$\frac{1}{N}\sum_{i}\int_{q_{i}}q\left(\theta_{i}\right)\log\frac{\Gamma \left(\sum_{k}\xi_{ik}\right)}{\prod_{k}\Gamma \xi_{ik}}\prod_{k}\theta_{ik}^{\xi_{ik}-1}d\theta -\sum_{\left(i,j\right)\in Edges}\frac{1}{d_{ij}}\left|\xi_{i}-\xi_{j}\right|$$

and simplify

1N∑i logΓ(∑kξik)∏kΓ(ξik)+∑i ∑k(ξik−1)Ψ(γik)−Ψ∑kγik︸cik−∑(i,j)∈Edges1dijξi−ξj

noting that term *C_ik_* does not depend on *ξ*.

(5) L(ξ)=1N∑i logΓ(∑kξik)∏kΓ(ξik)+∑i ∑kξikcik−∑(i,j)∈Edges1dijξi−ξj.

### 5.2. Alternating direction method of multipliers

In this section, we derive ADMM updates for maximizing objective [Equation (5)] efficiently.

We will denote the beta function (B), Gamma function (Γ), digamma function (ψ), and trigamma function (Φ)

$$B\left(\alpha \right)=\frac{\prod_{k}\Gamma \left(\alpha_{k}\right)}{\Gamma \left(\sum_{k}\alpha_{k}\right)}.$$

Using B our problem can be expressed as:

(6) minimizeξ1N∑i logB(ξi)−1N∑iξiTci+∑(i,j)∈Edges1dijξi−ξj1.

We add variables that will enable us to separate [Eq. (6)] into per-topic subproblems and convert the nonsmooth *l*1 penalty into constraints:

minimizeξ,τ,χ1N∑i logB(τi)−1N∑iτiTci+∑l1dlχl1

subject to τ=ξAξ=χ.

where **A** is a differencing matrix. We eliminate the norm using inequalities

minimizeξ,τ,χ1N∑i logB(τi)−1N∑iτiTci+∑l1dl1Tχl

(7) subject to τ=ξχ≽−Aξχ≽Aξ.

The augmented Lagrangian for Equation (7) is thus:

$$\begin{array}{cc} ℒ\left(\xi ,\chi ,u,v\right) & =\frac{1}{N}\sum_{i}\left[\log B\left(\tau_{i}\right)-\tau_{i}^{T}c_{i}\right]\\ & +\sum_{l}\frac{1}{d_{l}}1^{T}\chi_{l}-u_{1}^{T}\left(\chi -A\xi \right)-u_{2}^{T}\left(\chi +A\xi \right)\\ -v^{T}\left(\tau -\xi \right)+\rho ∕2{∥\tau -\xi ∥}_{2}^{2} \end{array}$$

### 5.2.1. Splitting the objective

We first list updates for different sets of variables and complete the square:

(8) e(k)=τ(k)+1∕ρv(k)ξ(k+1),χ(k+1),u(k+1)=argminξ,χ maxu ∑l1dl1Tχl−u1T(χ−Aξ)−u2T(χ+Aξ)+ρ∕2ξ−e(k)22.

(9) r(k)=ξ(k+1)−1∕ρv(k)+1∕ρcτ(k+1)=argminτ1N∑i logB(τi)+ρ∕2τ−r(k)22v(k+1)=v(k)+ρ(τ(k+1)−ξ(k+1)).

We solve for these updates in two parts:

- Fusion problem with Gaussian appearance [Eq. (8)].
- Dirichlet ML fitting with Gaussian regularization [Eq. (9)].

#### 5.2.2. Fusion problem with Gaussian appearance

In this section, we derive a primal-dual interior point optimization for solving [Eq. (8)]. We will solve for updates:

e(k)=τ(k)+1∕ρv(k)ξ(k+1),χ(k+1),u(k+1)=argminξ,χ maxu ∑l1dl1Tχl−u1T(χ−Aξ)−u2T(χ+Aξ)+ρ∕2ξ−e(k)22

using a primal-dual interior point method. We refer the reader to chapter 11 of Boyd and Vandenberghe (2004) for an overview of primal-dual interior point methods.

#### 5.2.3. Karush-Kuhn-Tucker (KKT) conditions for fusion problem

For simplicity, we introduce γ=(ξ,χ), and $C=\left[\begin{array}{cc} A & -I\\ -A & -I \end{array}\right]$ Letting

$$\begin{array}{cc} & f_{0}\left(\gamma \right)=\rho ∕2{∥\xi -e^{\left(k\right)}∥}_{2}^{2}+\sum_{l}\frac{1}{d_{l}}1^{T}\chi_{l}\\ & f_{1}\left(\gamma \right)=C\gamma \end{array}$$

Karush-Kuhn-Tucker (KKT) condition for the problem

(10) $$\begin{array}{c} \begin{array}{c} C\gamma ≼0 \end{array}\\ u≽0\\ \nabla_{\gamma }\left[f_{0}\left(\gamma \right)+u^{T}f_{1}\left(\gamma \right)\right]=0\\ u^{T}f_{1}\left(\gamma \right)=0 \end{array}$$

#### 5.2.4. Primal-dual updates for fusion problem

As in Boyd's book, Eq. 11.15, we will solve a modified KKT for the centering problem instead by replacing [Eq. (10)] with:

$$u^{T}f_{1}\left(\gamma \right)=\frac{1}{t}1$$

where 1/*t* will be tuned toward zero.

Following 11.7.1 in Boyd and Vandenberghe (2004), we state the modified KKT conditions in an equation form

$$r_{t}\left(\gamma ,u\right)=0$$

where

$$r_{t}\left(\gamma ,u\right)=\left[\begin{array}{c} \nabla f_{0}\left(\gamma \right)+Df_{1}{\left(\gamma \right)}^{T}u\\ -diag\left(u\right)f_{1}\left(\gamma \right)-1∕t1 \end{array}\right]$$

and derivative matrices given by

$$f\left(\gamma \right)=\left[\begin{array}{c} f_{1}\left(\gamma \right)\\ \cdots \\ f_{m}\left(\gamma \right) \end{array}\right],Df\left(\gamma \right)=\left[\begin{array}{c} \nabla f_{1}{\left(\gamma \right)}^{T}\\ \cdots \\ \nabla f_{m}{\left(\gamma \right)}^{T} \end{array}\right].$$

Calling out specific parts of *r*


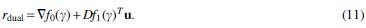

(12) $$r_{cent}=-diag\left(u\right)f_{1}\left(\gamma \right)-1∕t1.$$

To obtain Newton direction we solve the system

$$\left[\begin{array}{cc} \nabla^{2}f_{0}\left(\gamma \right)+u^{T}\nabla^{2}f_{1}\left(\gamma \right) & Df_{1}{\left(\gamma \right)}^{T}\\ -diag\left(u\right)Df_{1}\left(\gamma \right) & -diag\left(f_{1}\left(\gamma \right)\right) \end{array}\right]\left[\begin{array}{c} \Delta \gamma \\ \Delta u \end{array}\right]=-\left[\begin{array}{c} r_{dual}\\ r_{cent} \end{array}\right].$$

We are therefore interested in computing $\nabla f_{0}\left(\gamma \right)$, $\nabla^{2}f_{0}\left(\gamma \right)$, $\nabla^{2}f_{1}\left(\gamma \right)$, and $Df_{1}\left(\gamma \right)$. Recall that

$$\begin{array}{cc} \begin{array}{cc} \gamma & =\left(\xi ,\chi \right) \end{array}\\ C & =\left[\begin{array}{cc} A & -I\\ -A & -I \end{array}\right]\\ f_{0}\left(\gamma \right) & =\frac{1}{N}\sum_{i}\left[\log B\left(\xi_{i}\right)-\xi_{i}^{T}c_{i}\right]+\sum_{l}\frac{1}{d_{l}}1^{T}\chi_{l}\\ f_{1}\left(\gamma \right) & =C\gamma \end{array}$$

We can immediately observe that constraints are linear and hence $\nabla^{2}f_{1}\left(\gamma \right)=0$, and because *f_1_*(*γ*) = **C***γ*, we have *Df_1_*(*γ*) = **C**.

#### 5.2.5. Computing $\nabla f_{0}\left(\gamma \right)$

$$f_{0}\left(\gamma \right)=\rho ∕2{∥\xi -e^{\left(k\right)}∥}_{2}^{2}+\sum_{l}\frac{1}{d_{l}}1^{T}\chi_{l}$$

Hence,

$$\nabla_{\gamma }f_{0}=\left[\begin{array}{c} \rho \left(\xi_{1}-e_{1}^{\left(k\right)}\right)\\ \vdots \\ \rho \left(\xi_{N}-e_{N}^{\left(k\right)}\right)\\ \frac{1}{d_{1}}1_{K}\\ \vdots \\ \frac{1}{d_{L}}1_{K} \end{array}\right]$$

#### 5.2.6. Computing $\nabla^{2}f_{0}\left(\gamma \right)$

We observe overall structure of the matrix

$$\nabla^{2}f_{0}\left(\gamma \right)=\left[\begin{array}{cc} \nabla_{\xi }^{2}f_{0}\left(\xi ,\chi \right) & \nabla_{\xi }\nabla_{\chi }f_{0}\left(\xi ,\chi \right)=0\\ \nabla_{\xi }\nabla_{\chi }f_{0}\left(\xi ,\chi \right)=0 & \nabla_{\chi }^{2}f_{0}\left(\xi ,\chi \right)=0 \end{array}\right]$$

where blocks off-diagonal are zeros because of absence of cross-terms involving *χ* and *ξ*, and lower right block is zero because objective is linear in *χ*. Because

$$\nabla_{\xi }^{2}f_{0}\left(\xi ,\chi \right)=\rho I$$

we have

$$\nabla^{2}f_{0}\left(\gamma \right)=\left[\begin{array}{cc} \rho I & 0_{\left(N*K\right)\times \left(L*K\right)}\\ 0_{\left(L*K\right)\times \left(N*K\right)} & 0_{\left(L*K\right)\times \left(L*K\right)} \end{array}\right]$$

#### 5.2.7. Constructing and solving the linear system

Putting all the above pieces together,


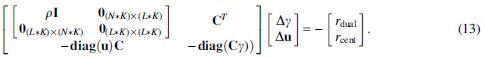


In practice, we solve the above linear system with a sparse linear solver to obtain step directions and perform a backtracking line search to determine step size.

#### 5.2.8. Dirichlet likelihood with Gaussian regularization

In this section, we solve for updates for optimizing [Eq. (9)]. Recall that we wish to solve for:

$$\tau^{\left(k+1\right)}={\mathrm{argmin}}_{\tau }\frac{1}{N}\sum_{i}\left[\log B\left(\tau_{i}\right)-\tau_{i}^{T}c_{i}\right]+\rho ∕2{∥\tau -t^{\left(k\right)}∥}_{2}^{2}$$

we observe that this problem is separable across *τ_i_*s

τi(k+1)=argminτili(τi)=argminτi logB(τi)−τiTci+ρ∕2τi−ti(k)22,

which can be simplified to

τi(k+1)=argminτi logB(τi)+ρ∕2τi−rik22,

where

$$r_{i}^{\left(k\right)}=t_{i}^{\left(k\right)}-\frac{1}{\rho }c_{i}$$

This can be accomplished using Newton's method.

#### 5.2.9. Matrix inversion-free Newton update for *τ*

To obtain a matrix-inversion-free Newton step, we use results from Minka (2000). Using the same notation as in Minka (2000). The gradient

$$\begin{array}{cc} & \tau^{new}=\tau^{old}-H^{-1}g\\ & y=\Psi \left(\sum_{k}\tau_{k}\right)\\ & g=\Psi \left(\tau \right)-1_{K}y+\rho \left(\tau -r\right) \end{array}$$

Hessian:

$$\begin{array}{cc} & z=\Phi \left(\sum_{k}\tau_{k}\right)\\ & Q=diag\left(\Phi \left(\tau \right)+\rho \right)\\ & H=Q+11^{T}z \end{array}$$

and derive the update